# Insights into the Metabolomic Diversity of *Latilactobacillus sakei*

**DOI:** 10.3390/foods11030477

**Published:** 2022-02-06

**Authors:** Federica Barbieri, Luca Laghi, Chiara Montanari, Qiuyu Lan, Alessia Levante, Fausto Gardini, Giulia Tabanelli

**Affiliations:** 1 Department of Agricultural and Food Sciences, University of Bologna, 47521 Cesena, Italy; federica.barbieri16@unibo.it (F.B.); chiara.montanari8@unibo.it (C.M.); qiuyu.lan@unibo.it (Q.L.); fausto.gardini@unibo.it (F.G.); 2 Department of Food and Drug, University of Parma, 43121 Parma, Italy; alessia.levante@unipr.it; 3 Department of Agricultural and Food Sciences, University of Bologna, 40127 Bologna, Italy; giulia.tabanelli2@unibo.it

**Keywords:** *Latilactobacillus sakei*, carbon sources, amino acid metabolism, metabolomics, ^1^H-NMR

## Abstract

*Latilactobacillus sakei* (*L. sakei*), widely used as a starter culture in fermented sausages, is a species adapted to meat environments. Its ability to survive for a long time in such products is due to the exploitation of different metabolic pathways to gain energy (hexose and pentose sugar fermentation, amino acids catabolism, etc.). Since *L. sakei* demonstrates high phenotypic and metabolic strain biodiversity, in this work, a metabolomic approach was used to compare five strains of different origins. They were cultivated in a defined medium with glucose or ribose at two concentrations, and analyzed through nuclear magnetic resonance (^1^H-NMR) spectroscopy to monitor amino acid consumptions and accumulation of organic acids and aroma compounds. The results showed that all the strains were able to use arginine, especially when cultivated with ribose, while serine was consumed mainly in the presence of glucose. Aroma compounds (i.e., diacetyl and acetoin) were mainly accumulated in samples with ribose. These aspects are relevant for starter cultures selection, to confer specific features to fermented sausages, and to optimize the fermentations. Moreover, the use of ^1^H-NMR allowed the fast identification of different classes of compounds (without derivatization or extraction procedures), providing a powerful tool to increase the knowledge of the metabolic diversity of *L. sakei*.

## 1. Introduction

*Latilactobacillus sakei* (*L. sakei*) is a rod-shaped lactic acid bacterium (LAB) which is highly adapted to meat and fish environments, where it rapidly grows, competing with the other species, which are common components of the microbiota. In particular, *L. sakei* is often the dominant species in spontaneously fermented sausages [1,2], even after long ripening times (several weeks or months) in the absence of fermentable sugars. In these harsh conditions, it takes advantage of the high protein availability (the species is auxotrophic for 18 amino acids), its ability to grow at a high NaCl concentration, and its resistance to oxidative stress, enhanced by the presence of iron and heme in meat [3,4]. In addition, the species can efficiently grow at low temperatures (20 °C and below), such as those characterizing the fermentation and ripening of Mediterranean-type sausages [5]. The competitiveness of this species is also assured by the efficiency of using the few carbon sources available for energy production, and the possibility of synthetizing bacteriocins such as sakacins and lactocins [6,7,8].

Because of these characteristics, strains of *L. sakei* are widely used as starter cultures in industrial meat fermentations [9], with the aim to inhibit the growth of pathogenic, as well as spoilage, microorganisms. 

Studies of *L. sakei* have revealed that its genome is relatively small, ranging between 1.8 and 2.3 Mbp, with a difference of up to 25% in its content, and these differences may involve approximatively 500 genes [4,10,11]. These genes (or genomic islands) affect cell surface, carbon metabolism, regulatory functions, bacteriocin production, and adaptation to redox variation [12]. In other words, the ability of strains to adapt to different environmental and stress conditions may be extremely variable.

During sausage fermentation, *L. sakei* can colonize the habitat throughout the ripening period, avoiding the growth of undesired microorganisms. Its persistence in the habitat depends on its ability to produce metabolic energy, even when the hexoses, which are fermented through the homofermentative pathway, are completely depleted.

Among the alternative pathways for energy production, one well known pathway is the ability of this species to ferment pentoses contained in nucleosides via the phosphoketolase pathway [11,13]. In addition, the arginine deiminase (ADI) pathway is active in *L. sakei*, even with a different efficiency, giving it a competitive advantage in matrices with high arginine content, such as meat [14,15]. Alternative energy sources in the absence of fermentable sugars can be derived from other amino acids, even if *L. sakei* lacks transaminases [10]. Threonine [14] and cysteine [15] are depleted in remarkable amounts by this species during growth, under defined conditions. In addition, the increased production of serine dehydratase, responsible for the deamination of serine and the production of pyruvate, was observed in *L. sakei* on specific nutritional conditions [14,15]. In addition, the presence of a gene coding for L-threonine dehydrogenase has been described in some *L. sakei* strains [14].

The metabolism of this species in environments which are lacking in fermentable sugars is also explained by an efficient pyruvate metabolism carried out for the generation ATP and the balance of reducing power (regeneration of NAD+). The genetics and the transcriptomics of the pyruvate formate lyase (PFL) pathway, which leads to the accumulation of formate, acetate, and ethanol in anaerobic or reducing conditions, have already been studied in *L. sakei*. In addition, this species is characterized by the pyruvate oxidase (POX) pathway and the pyruvate dehydrogenase complex (PDC), active in aerobic conditions, of which the final products are CO_2_, acetate, and acetoin [3,16]. These strategies for facing stressful nutritional conditions have important consequences for the competitiveness of the species in the habitat, and on the formation of the sensorial profile of sausages. The variability of the metabolic response of *L. sakei* has already been highlighted by proteomic and transcriptomic studies [3,16]. 

In this study, a metabolomic approach has been adopted. Five *L. sakei* strains (including the type strain DSMZ 20017t) were grown on defined media containing amino acids as nitrogen sources and glucose or ribose as a fermentable substrate. The two sugars were added to culture media in two amounts: 2.5 mM and 25 mM. The consumption or accumulation of amino acids and the production of metabolites were monitored with the ^1^H-NMR protocol proposed by Barbieri et al. [17]. The aim was to assess the variability in the strain responses with respect to the metabolism of the molecules relevant for *L. sakei* growth, and the potential impact on industrial meat products, such as fermented sausages.

## 2. Materials and Methods

### 2.1. Microbial Strains 

Five strains belonging to the species *Latilactobacillus sakei* were used in this study. In detail, they were the type strain DSMZ 20017t (DSMZ, Braunschweig, Germany), the commercial strain Chr82 used as a starter culture in fermented cured meats, provided by Chr. Hansen (Parma, Italy), and three strains from the collection of the Department of Agricultural and Food Sciences (University of Bologna). Among the latter, the strains BR3 and TA13 were isolated from spontaneously fermented pork sausages produced in the Emilia Romagna region, while the strain CM3 was isolated from dried camel meat produced in Algeria. These strains have already been characterized for some phenotypic features [11]. Strains were maintained in de Man Rogosa and Sharp (MRS) medium (Oxoid, Basingstoke, UK) with 20% (*w*/*v*) glycerol at −80 °C, until usage.

### 2.2. Growth Media

To evaluate the effect of different carbon sources on *L. sakei* growth, the strains were grown in MRS broth, and prepared by weighing the single components (except for glucose) according to Oxoid formulation. Glucose (4.5 g/L) or ribose (3.75 g/L) was added as a carbon source, so that the final concentration in the medium was 25 mM. Moreover, in the medium with ribose, a small amount of glucose (0.2 g/L) was also added to stimulate microbial growth in the initial phase, according to McLeod et al. [11]. Then, cells grown overnight at 30 °C under statically microaerophilic conditions in these two media were collected by centrifugation (10,000 rpm for 10 min), washed with sterile physiological solution (0.9%, *w*/*v* NaCl), and resuspended at a cell concentration of about 6.5 log CFU/mL in a defined medium (DM), containing macro components, vitamins, nucleotides, and amino acids, as reported by Barbieri et al. [17]. In particular, 0.2 g/L of each amino acid was added to the medium, to reach the following concentrations (expressed as mM): alanine (ala) 2.24, arginine (arg) 1.15, asparagine (asg) 1.52, aspartic acid (asp) 1.50, cysteine (cys) 1.65, glutamic acid (glu), glutamine (glm) 1.36, glycine (gly) 2.66, histidine (his) 1.29, isoleucine (ile) 1.52, leucine (leu) 1.52, lysine (lys) 1.37, methionine (met) 1.34, phenylalanine (phe) 1.21, proline (pro) 1.74, serine (ser) 1.90, threonine (thr) 1.68, tryptophan (try) 0.98, tyrosine (tyr) 1.10, valine (val) 1.71. When it comes to carbon sources, cells grown in modified MRS with 25 mM of glucose were suspended in DM with 2.5 mM (2.5 G) or 25 mM (25 G) of glucose, while cells grown in modified MRS with 25 mM of ribose were suspended in DM with 2.5 mM (2.5 R) or 25 mM (25 R) of ribose. The medium was previously sterilized by filtration at 0.22 µm (Sartorius Lab Instruments GmbH & Co. KG, Göttingen, Germany). Its initial pH was 6.50 ± 0.03. The different samples were incubated at 30 °C and analyzed after 24 and 48 h.

### 2.3. Microbiological Analyses and pH

The microbial counts of the five *L. sakei* strains grown in the different conditions were carried out by preparing serial decimal dilutions in sterile physiological solution, and then plating them onto MRS agar (Oxoid, Basingstoke, UK). The plates were then incubated at 30 °C for 48 h. 

The pH was measured in triplicate with a pHmeter Basic 20 (Crison, Modena, Italy).

### 2.4. Untargeted Metabolomics Analysis by ^1^H-NMR

By following Barbieri et al. [17], we created a ^1^H-NMR analysis solution, constituted by a 10 mM D_2_O solution of 3-(trimethylsilyl)-propionic-2,2,3,3-d4 acid sodium salt (TSP) as a chemical-shift reference. A 1 M phosphate buffer granted a pH of 7.00 ± 0.02, while 10 μL of NaN_3_ 2 mM avoided microbial proliferation. We prepared the samples of growth medium for ^1^H-NMR by thawing and centrifuging 1 mL of each for 15 min at 18,630 g and 4 °C. We then added 200 μL of ^1^H NMR analysis solution to 700 μL of supernatant, centrifuging once more at the above conditions before analysis.

We recorded the ^1^H-NMR spectra at 298 K with an AVANCE III spectrometer (Bruker, Milan, Italy), which operates at a frequency of 600.13 MHz, and is equipped with the software Topspin (ver. 3.5). Following Barbieri et al. [17], we employed the first increment of the pulse sequence designed to evidence the nuclear overhauser effect (NOESY), enriching it with a spoil gradient to suppress the HOD residual signal. For each spectrum, we summed up 256 transients, covering a spectral window of 7184 Hz, with 32 K data points. We employed the scripts of Topspin to adjust the phase and baseline of each spectrum, as well as to calculate the signal-to-noise ratio. We performed a signal assignment via a comparison with the spectra of pure compounds by Chenomx software (Chenomx Inc., Canada, ver 8.3) with Chenomx (ver. 10) and HMDB (release 2) libraries. The concentration of each molecule was calculated from the area of one of its signals, measured by global spectra deconvolution, implemented in MestReNova software (Mestrelab research S.L. Santiago De Compostela (Spain)-ver 14.2.0-26256). This was done after applying a line broadening of 0.3 and a baseline adjustment by the Whittaker Smoother procedure. 

### 2.5. Statistical Analysis

An ANOVA was carried out to highlight significant differences (*p* < 0.05) among cell counts and pH variation.

A principal component analysis (PCA), according to the type of data, was carried out based on correlation matrix (accumulation of metabolites) or covariance matrix (percentage variation of amino acids). 

The statistical package Statistica 8 (StatSoft Inc., Tulsa, OK, USA) was used for the analyses.

## 3. Results

### 3.1. Microbial Counts and pH

All the strains were inoculated in the defined medium (DM), of which the pH was 6.50, at an initial mean concentration of about 6.6 log CFU/mL. For each condition, the differences of cell concentrations, with respect to the initial inoculum and the pH variations after 24 and 48 h of fermentation at 30 °C, are reported in Table 1.

In general, after 24 h, all the strains showed minor growth in media with sugars at 2.5 mM. The strains Chr82 and DSMZ 20017t increased by less than 1 log unit. On the other hand, the strains TA13 and BR3 were characterized by higher increases, comparable with the data observed with sugars at 25 mM. Interestingly, all the strains grown on ribose 2.5 mM showed higher increases with respect to the performances in the presence of glucose 2.5 mM.

After 24 h, when sugars were present at a higher concentration, the type strain DSMZ 20017t presented a lower increase (less than 1 log unit). Concerning the other strains, cell concentration increased by between 1.03 and 1.86 log CFU/mL. An exception was represented by the strain Chr82 grown on ribose 25 mM, which increased by only 0.87 log CFU/mL. 

After 48 h, cell viability was lower than the one at 24 h. For the strains Chr82 and DSMZ 20017t, in all the conditions, the viability was lower than the initial inoculum. Anyway, relevant decreases were also observed for the other strains. This was not unexpected, since the rapid loss of viability of *L. sakei* grown on culture media has already been observed [17,18].

The pH measured after 24 h was systematically lower in the samples containing glucose. In the media containing 2.5 mM glucose, the pH decrease (ΔpH) ranged between −0.65 (CM3) and −0.79 (TA13), while in 2.5 mM ribose, ΔpH varied between −0.38 (BR3) and −0.66 (DSMZ 20017t). In the media added to 25 mM of sugar, the pH decrease was more evident, with ΔpH values ranging between −2.57 (Chr82) and −2.65 (TA13) for glucose and −2.00 (TA13) and −2.26 (DSMZ 20017t) for ribose. After 48 h, the pH did not substantially change, except for TA13 grown on 25 mM ribose, in which a further decrease of approx. 0.2 was observed.

According to these data, the maximum growth, corresponding to the lowest pH reached, was attained after 24 h, with the exception of the strain TA13. For this reason, the subsequent metabolomic analyses were carried out on the samples incubated for 24 h only.

### 3.2. Metabolomic Analyses

#### 3.2.1. Amino Acid Variation

**Figure 1:**
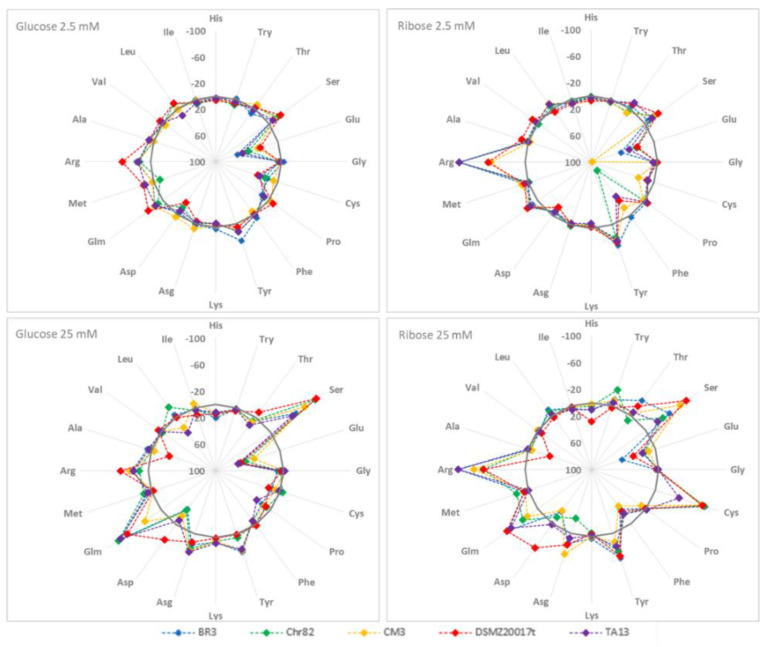
Percentage variation with respect to the initial concentration of amino acids after 24 h of incubation of the five *L. sakei* strains in the defined media with different sugar amounts. Negative values mean amino acid consumption, and positive values mean amino acids accumulation, while the continuous grey line represents the interface (0%).

Figure 1 summarizes the amino acid relative variations in the DM under different nutritional conditions after 24 h of fermentation.

With glucose 2.5 mM, glutamate is the only amino acid showing relevant increases (from +65.4% to +28.9% in BR3 and DSMZ20017t, respectively). The type strain DSMZ20017t also showed a maximum increase in the concentration of aspartate (+22.6%). The main decreases involved serine (from −22.1% to −6.4% in DSMZ20017t and BR3, respectively), glutamine, and arginine. For these latter two amino acids, the most relevant decreases concerned DSMZ20017t (−28.1 and −43.0%, respectively). 

With ribose 2.5 mM, overall glutamate was once more the amino acid showing the highest increase (especially in CM3), together with phenylalanine, especially for Chr82 (+84.1%). In contrast, relevant decreases were observed for arginine: −100% in BR3, Chr82, and TA13, while the diminutions were −52.4% and −55.8% in CM3 and DSMZ20017t, respectively.

All the strains presented marked decreases of serine when grown in the medium containing glucose 25 mM. In detail, this amino acid decreased from −40.6% (TA13) to −84.5% (DSMZ20017t). In addition, glutamine showed relevant decreases (between −62% and −76%), with the exception of the strain CM3. Under these conditions, arginine decreased only marginally. In contrast, glutamate and aspartate increased, as already observed in the strains grown with the same sugar at 2.5 mM concentration.

The presence of ribose 25 mM affected the trends of specific amino acids. Phenylalanine increased in all the strains in correspondence with a diminution of tyrosine. In addition, arginine drastically decreased by up to −100% in BR3 and TA13, while in the remaining strains, its concentration ranged from −61.8% (DSMZ20017t) to −76.4% (CM3). Cysteine showed a relevant decrease (−70% and more) in all the strains, with the exception of TA13 (−37.7%). Serine and glutamate presented behaviors similar to those observed in the samples with 25 mM glucose.

**Figure 2:**
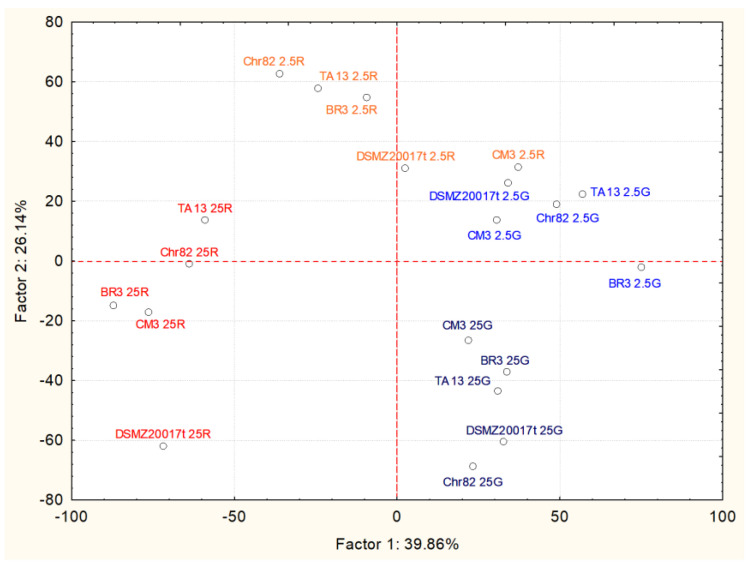
Results of PCA based on covariance matrix of percentages of amino acid variations after 24 h of incubation for the five *L. sakei* strains in the defined media with different sugar amounts. Projection of case coordinates on the sample score plot of the first two factors, explaining 66% of variability.

The relationships between amino acid variations and the type and amount of available sugars have been further explored through PCA analysis. The results concerning the first two factors, explaining 66.0% of total samples variability, are reported in Figure 2. According to the factor loadings of the covariance analysis (Table 2), the major positive contribution to the first factor was represented by cysteine (0.8737) and arginine (0.8125), while phenylalanine was the major negative contributor (−0.6023). Factor 2 was mainly affected by the positive values of serine (0.8619), glutamine (0.8150) and lysine (0.7236), and by the negative values of histidine (−0.7889).

The strains resulted in being well grouped according to the type and amount of carbon sources added to the growth medium used for their cultivation. Interestingly, the cluster formation was determined by few amino acids. Among them, arginine, consumed to different extents in almost all conditions, was the most important, with a peculiar effect of discriminating cells grown on glucose from those grown on ribose. Generally, the presence of ribose determined the highest consumption, or even the complete depletion, of this amino acid. The importance of the arginine deiminase pathway (ADI) in *L. sakei* is well known. ADI allows an improved tolerance in acidic stress and generates energy (ATP) when fermentation sugars are depleted, allowing the survival and metabolic activity of *L. sakei* throughout the ripening process [19]. The operon responsible for this pathway (arc operon) comprises three main enzymes (arginine deiminase, catabolic ornithine carbamoyl transferase, and carbamate kinase), but other genes coding for other functions (such as antiporters) may be present. It is an inducible operon subjected to glucose repression and induced by arginine. Anaerobiosis stimulates its activity, and the optimum pH is 6 [20]. In this study, the absence of glucose increased the efficiency of ADI in metabolizing arginine. An increase in the transcription of arginine deiminase in *L. sakei* 23K (but not in the other two *L. sakei* strains) during the growth on ribose was already described by McLeod et al. [16]. Montanari et al. [15] reported a similar influence of ribose on ADI in the resting cells of *Lat. sakei* strains.

Cysteine contributed to the separation of the samples containing the highest content of glucose and ribose. In fact, it decreased when *L. sakei* strains were grown in the medium containing ribose 25 mM, but not in the presence of glucose. Information on cysteine catabolism in *L. sakei* and LAB is scarce. In general, Smacchi and Gobbetti [21] reported that in LAB, cystathionine-γ-lyase may degrade cystine to ammonia, hydrogen sulfide, and pyruvate. This latter molecule can follow several alternative pathways in order to produce ATP, and control the redox balance inside the cells [16].

The variation of serine was important in discriminating the samples grown on media containing sugars at 25 mM from those containing the same sugar at 2.5 mM. Serine utilization is important for survival during the stationary phase, and its catabolism increases the pool of pyruvate [22]. McLeod et al. [14] observed a relevant utilization of serine in *L. sakei* strains grown under glucose limiting conditions, indicating that the consumption of this amino acid began when the cells depleted the fermentable sugars and entered the stationary phase. 

Moreover, glutamine consumption (higher in the samples grown on media with sugars at 25 mM) contributed to the separation between the groups. Glutamine may be transformed in glutamate (via glutaminase), which increased in the same samples, with the liberation of NH_3_, or its conversion into pyroglutamate [23].

Less clear is the increase of phenylalanine in the samples containing ribose 2.5 mM. Its increase is compensated by a concomitant reduction of tyrosine. Latorre-Moratalla et al. [24] observed the relevant presence of phenylalanine in sausages fermented with a *L. sakei* strain, but they attributed this concentration to the peptidase activity of the strain on meat proteins.

#### 3.2.2. Accumulation of Other Metabolites

In addition to the variation of amino acids in different conditions, the ^1^H-NMR analysis allowed the detection of several compounds produced by the metabolism of *L. sakei* strains (Table 3). 

Sugars were completely depleted after 48 h of fermentation in the media containing 2.5 mM of glucose and ribose. In the medium containing 25 mM of glucose, residues of this sugar were found in the media fermented by Chr82 (2.45 mM), CM3 (0.44 mM), TA13 (0.43 mM), and BR3 (0.38 mM). When ribose was added at its maximum concentration, residues were found in the media fermented by Chr82 (2.11 mM) DSMZ20017t (0.31 mM), CM3 (0.30 mM), BR3 (0.21 mM), and especially in TA13 (6.90 mM).

**Figure 3:**
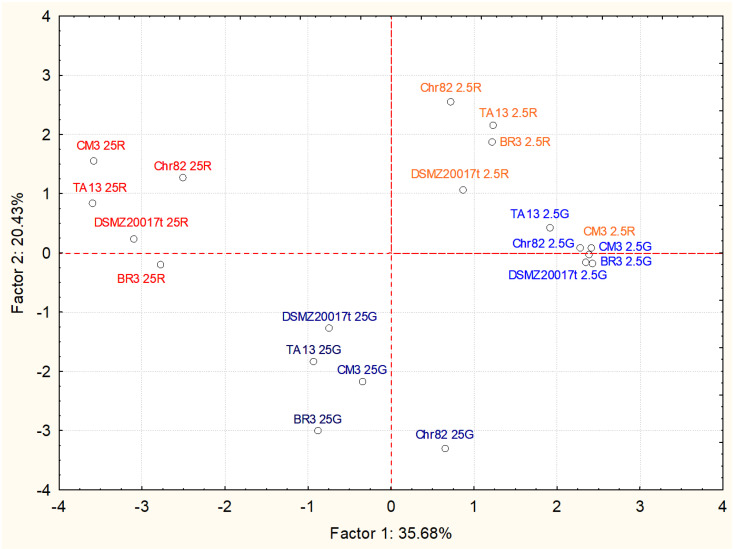
Results of PCA based on correlation matrix of metabolites accumulated after 24 h of incubation of the five *L. sakei* strains in the defined media with different sugar amounts. Projection of case coordinates on the sample score plot of the first two factors, explaining about 56% of variability.

The role of the remaining sugars and metabolites, accumulated as a result of the anabolic pathways of *L. sakei*, was investigated through a PCA based on correlations. The results, reported in Figure 3, showed that the four groups, corresponding to the different growth media, were well separated, except for CM3 grown on ribose 2.5 mM, which clustered with the samples 2.5 G. 

The factor 1 (35.68% of the variability) separated the 25G from the 25R driven by the contribution of acetate (factor loading −0.8773), diacetyl (−0.8770) and, to a lesser extent, ethanol (−0.6928) and pyruvate (−0.6218), as reported in Table 4. Factor 2 (20.43% of variability) contributed to the discrimination of the sample containing ribose from the sample containing glucose. In particular, ornithine (loading factor 0.6543) and acetoin (0.5419) grouped the ribose samples in the upper part of the plot, while lactate (−0.7815) contributed to the grouping of the glucose samples in the lower part.

The alternative pathways involving pyruvate when fermentable sugars are scarce or depleted (mixed-acid fermentation) are well studied [25]. Pyruvate formate lyase (PFL) and pyruvate oxidase (POX) are key enzymes in this perspective, in the absence or presence of oxygen, respectively. The accumulation of products such as formate, acetate, and ethanol is the consequence of these pathways. It is interesting to observe that these molecules contribute to the separation of the samples containing ribose from those containing glucose. In other words, the presence of ribose seems to stimulate alternative metabolic pathways of pyruvate. Increases in the transcription of genes involved in these pathways (pyruvate oxidase, pyruvate dehydrogenase and pyruvate formate lyase) were observed in *L. sakei* grown on ribose if compared with those grown on glucose [16]. These pathways are also favored by metabolic routes producing pyruvate, such as serine dehydratase, which can become crucial in the survival strategy when sugars are completely depleted [14]. Pyruvate can be directed toward the formation of acetoin and diacetyl, through the enzyme α-acetolactate synthase. In particular, the production of acetoin resulted in being higher in the sample containing ribose, while diacetyl discriminates between the samples grown with a higher sugar supply (25 mM). Interestingly, the accumulation of diacetyl in response to cultivation on high ribose concentration might be due to increased acetoin reductase (EC: 1.1.1.304) activity. The conversion of acetoin to diacetyl, indeed, could be involved in the regeneration of reduced NAD(P)H+ cofactor pool, as shown for other lactic acid bacteria [26]. Finally, ornithine was detected only in the sample grown on ribose, and correlated to the consumption of arginine. 

## 4. Conclusions

The present metabolomics investigation highlighted different behaviors among *L. sakei* strains in relation to the quantitative and qualitative presence of fermentable sugars in the growth media, and to the different capacity to metabolize amino acids. The use of ^1^H-NMR provided a powerful tool to investigate the metabolic diversity on starter cultures, as it is a fast method to identify the different classes of compounds involved in cell metabolisms. 

All the strains were able to use arginine, especially in the presence of sugar limitation. Nevertheless, the ability to metabolize this amino acid-activating ADI pathway was strongly influenced by the sugar present in the medium, being higher in samples containing ribose. On the contrary, serine was strongly consumed mainly in the presence of glucose.

Other metabolic products, such as diacetyl and acetoin, which can affect the sensory profile of fermented sausages, were mainly accumulated in samples without glucose, indicating a higher activation of POX and PFL, under these conditions. Future perspectives include an in-depth analysis of the transcriptional activation of the metabolic pathways involved in the production of these compounds, to obtain a clearer description of the drivers of metabolic diversity in *L. sakei*. 

These aspects are relevant for the selection of starter cultures to obtain tailor-made strains able to confer specific features to the fermented sausages and to optimize the fermentation process. Indeed, besides their persistence throughout production and commercialization via the activation of mechanisms for the adaptation to food environment, such as toxin–antitoxin systems, more attention is now devoted to the physiological and technological properties able to improve industrial performance, contributing to the recognizability of the final products.

## Figures and Tables

**Table 1:** Cell counts and pH values observed during the incubation of the five *L. sakei* strains in the different DM. Data are expressed as variation with respect to the initial values (also reported in the table).

Strains	DM	Cell Counts (log CFU/mL)			pH		
		T0	Δlog 24 h	Δlog 48 h	T0	ΔpH 24 h	ΔpH 48 h
Chr82	2.5 G	6.68 ± 0.05	0.71 ^Aa^* ± 0.05	−0.28 ^aA^ ± 0.04	6.48 ± 0.01	−0.75 ^aA^ ± 0.04	−0.71 ^aA^ ± 0.02
	2.5 R	6.80 ± 0.04	0.82 ^aA^ ± 0.11	−0.90 ^aB^± 0.08	6.47 ± 0.03	−0.46 ^aB^ ± 0.02	−0.47 ^aB^ ± 0.03
	25 G	6.68 ± 0.05	1.77 ^aB^± 0.23 ^b^	−0.58 ^aC^ ± 0.10	6.50 ± 0.01	−2.57 ^aC^ ± 0.05	−2.62 ^aC^ ± 0.02
	25 R	6.80 ± 0.04	0.87 ^aA^ ± 0.09	ND **	6.47 ± 0.02	−2.13 ^aD^ ± 0.02	−2.17 ^aD^ ± 0.02
DSMZ 20017t	2.5 G	6.69 ± 0.03	0.16 ^bA^ ± 0.05	ND	6.48 ± 0.01	−0.75 ^aA^ ± 0.03	−0.77 ^aA^± 0.02
	2.5 R	6.54 ± 0.04	0.63 ^aB^ ± 0.07	−0.60 ^bA^± 0.12	6.47 ± 0.03	−0.66 ^bB^ ± 0.05	−0.66 ^bB^ ± 0.02
	25 G	6.69 ± 0.03	0.65 ^bB^ ± 0.09	0.09 ^bB^ ± 0.03	6.50 ± 0.01	−2.59 ^aC^ ± 0.03	−2.60 ^aC^ ± 0.04
	25 R	6.54 ± 0.04	0.98 ^aB^ ± 0.22	−0.60 ^aA^ ± 0.12	6.47 ± 0.02	−2.26 ^bD^ ± 0.02	−2.25 ^bD^ ± 0.02
CM3	2.5 G	6.75 ± 0.03	0.83 ^aA^ ± 0.08	−0.27 ^aA^ ± 0.03	6.48 ± 0.01	−0.65 ^bA^ ± 0.04	−0.61 ^bA^ ± 0.03
	2.5 R	6.70 ± 0.02	1.21 ^bB^ ± 0.28	0.85 ^cB^ ± 0.04	6.47 ± 0.03	−0.41 ^aB^ ± 0.03	−0.44 ^aB^ ± 0.02
	25 G	6.75 ± 0.03	1.48 ^aB^ ± 0.31	0.87 ^cB^ ± 0.08	6.50 ± 0.01	−2.62 ^aC^ ± 0.03	−2.59 ^aC^ ± 0.04
	25 R	6.70 ± 0.02	1.32 ^bB^ ± 0.24	0.48 ^bC^ ± 0.10	6.47 ± 0.02	−2.24 ^bD^ ± 0.02	−2.22 ^bD^ ± 0.02
TA13	2.5 G	6.54 ± 0.06	1.33 ^cA^ ± 0.11	0.13 ^bA^ ± 0.03	6.48 ± 0.01	−0.79 ^aA^ ± 0.02	−0.76 ^aA^ ± 0.02
	2.5 R	6.63 ± 0.04	1.43 ^cA^ ± 0.20	0.80 ^cB^ ± 0.05	6.47 ± 0.03	−0.48 ^aB^ ± 0.02	−0.46 ^aB^ ± 0.03
	25 G	6.54 ± 0.06	1.03 ^cB^ ± 0.09	0.26 ^dC^ ± 0.02	6.50 ± 0.01	−2.65 ^aC^ ± 0.04	−2.63 ^aC^ ± 0.03
	25 R	6.63 ± 0.04	1.86 ^cC^ ± 0.25	0.32 ^bC^ ± 0.05	6.47 ± 0.02	−2.00 ^cD^ ± 0.04	−2.23 ^bD^ ± 0.03
BR3	2.5 G	6.64 ± 0.03	1.38 ^cA^ ± 0.13	0.13 ^bA^ ± 0.02	6.48 ± 0.01	−0.71 ^aA^ ± 0.05	−0.67 ^aA^ ± 0.02
	2.5 R	6.62 ± 0.02	1.60 ^cB^ ± 0.20	1.26 ^dB^ ± 0.08	6.47 ± 0.03	−0.38 ^cB^ ± 0.02	−0.38 ^cB^ ± 0.03
	25 G	6.64 ± 0.03	1.22 ^aA^ ± 0.11	−0.34 ^eC^ ± 0.03	6.50 ± 0.01	−2.59 ^aC^ ± 0.03	−2.59 ^aC^ ± 0.04
	25 R	6.62 ± 0.02	1.83 ^cC^ ± 0.32	0.33 ^bD^± 0.02	6.47 ± 0.02	−2.21 ^bD^ ± 0.02	−2.22 ^bD^ ± 0.05

* the presence of lowercase letters indicates significant differences (in Δlog or ΔpH after 24 or 48 h) of the same condition between the five strains, while capital letters indicate differences between the growth conditions (i.e., 2.5 G, 2.5 R, 25 G, 25 R) for the same strain. ** ND: not determined, corresponding to a decrease higher than 3 log CFU/mL.

**Table 2:** Factor loadings of the first two factors of PCA (accounting for 66% of the total variability).

Amino Acid	Factor 1	Factor 2
Histidine	−0.1238	−0.7889
Tryptophane	0.6167	0.1225
Threonine	0.5234	−0.0476
Serine	0.2388	0.8619
Glutamate	0.5965	−0.1495
Glycine	−0.1867	−0.0860
Cysteine	0.8737	0.3374
Proline	0.3787	−0.5586
Phenylalanine	−0.6023	0.3606
Tyrosine	0.4433	−0.1023
Lysine	−0.1854	0.7236
Asparagine	0.0910	0.6615
Aspartate	0.1229	0.2250
Glutamine	0.0948	0.8150
Methionine	0.1111	0.0362
Arginine	0.8125	−0.4604
Alanine	−0.3494	−0.5333
Valine	−0.3874	−0.3328
Leucine	0.1979	−0.1536
Isoleucine	0.0593	0.1395

**Table 3:** Concentration (expressed as mM) of some metabolites detected by ^1^H-NMR after 24 h of incubation of the five *L. sakei* strains in the different DM.

Strains	DM *	Lactate	Acetate	Ribose	Glucose	Pyruvate	Acetoin	Diacetyl	Ethanol	1,3 Dihydroxy Acetone	Ornithine	Pyro Glutamate	Formate	Fumarate
Chr82	2.5 G	4.14	1.62	- **	-	-	0.03	-	0.03	-	-	0.04	0.01	-
	2.5 R	0.79	2.20	-	-	-	0.34	0.17	0.04	-	0.69	0.10	0.04	-
	25 G	42.33	3.74	-	2.45	-	0.11	0.03	0.04	0.03	-	0.02	-	0.01
	25 R	19.19	21.44	2.11	-	0.06	0.21	0.30	0.04	0.02	0.28	0.04	0.04	0.02
DSMZ20017t	2.5 G	3.89	0.34	-	-	-	-	-	0.03	-	-	0.04	-	-
	2.5 R	0.92	1.52	-	-	-	0.15	0.09	0.04	-	-	0.09	0.10	-
	25 G	43.35	4.83	-	-	-	-	0.04	0.05	0.05	-	0.11	0.10	0.02
	25 R	24.79	26.16	0.31	-	0.01	0.10	0.31	0.05	0.05	-	0.12	0.10	0.03
CM3	2.5 G	3.57	1.63	-	-	-	0.05	0.02	0.02	-	-	0.00	0.01	-
	2.5 R	1.57	0.88	-	-	-	0.05	0.02	0.03	-	0.05	0.01	0.01	-
	25 G	38.54	1.48	-	0.44	-	0.02	0.13	0.06	0.06	-	0.06	0.01	0.01
	25 R	18.93	22.39	0.30	-	0.02	0.41	0.57	0.06	0.06	0.23	0.06	0.10	0.01
TA13	2.5 G	3.51	1.43	-	-	-	0.04	0.01	0.03	-	-	0.09	0.01	-
	2.5 R	1.23	3.00	-	-	-	0.11	0.06	0.04	-	0.90	0.11	0.04	-
	25 G	46.58	3.49	-	0.43	-	-	0.10	0.05	0.03	-	0.06	0.12	0.03
	25 R	17.40	14.75	6.90	-	0.01	0.05	0.33	0.06	0.02	0.37	0.10	0.10	0.06
BR3	2.5 G	3.87	0.90	-	-	-	0.03	-	0.03	-	-	0.02	-	-
	2.5 R	1.39	2.90	-	-	-	0.11	0.07	0.04	-	0.10	0.07	0.04	-
	25 G	43.27	5.67	-	0.38	0.01	0.06	0.07	0.10	0.05	-	0.01	-	0.02
	25 R	19.82	17.82	0.21	-	0.05	0.16	0.30	0.08	0.08	0.43	0.03	-	0.02

* Defined medium with 2.5 mM (2.5 G) or 25 mM (25 G) of glucose, or 2.5 mM (2.5 R) or 25 mM (25 R) of ribose. ** under the detection limit (0.01 mM).

**Table 4:** Factor loadings of the first two factors of PCA (accounting for about 56% of the total variability).

Compound	Factor 1	Factor 2
Fumarate	−0.7609	−0.2549
1,3 dihydroxy acetone	−0.7428	−0.4562
Lactate	−0.5128	−0.7815
Ribose	−0.5056	0.1840
Glucose	0.0302	−0.6402
Pyruvate	−0.6218	0.1667
Acetoin	−0.4050	0.5419
Ethanol	−0.6923	−0.4185
Diacetyl	−0.8770	0.3222
Ornithine	−0.0828	0.6543
Acetate	−0.8773	0.1989
Pyroglutamate	−0.3227	0.4526
Formate	−0.5544	0.2356
